# Relationship between Posture and Non-Contact Lower Limb Injury in Young Male Amateur Football Players: A Prospective Cohort Study

**DOI:** 10.3390/ijerph18126424

**Published:** 2021-06-14

**Authors:** Suzanne J. Snodgrass, Kathleen E. Ryan, Andrew Miller, Daphne James, Robin Callister

**Affiliations:** 1 School of Health Sciences, The University of Newcastle, Callaghan, NSW 2308, Australia; ryankathleen27@gmail.com (K.E.R.); daphne.james@newcastle.edu.au (D.J.); 2 Priority Research Centre for Physical Activity and Nutrition, The University of Newcastle, Callaghan, NSW 2308, Australia; Robin.Callister@newcastle.edu.au; 3 School of Education, The University of Newcastle, Callaghan, NSW 2308, Australia; Andrew.Miller@newcastle.edu.au; 4 School of Biomedical Sciences and Pharmacy, The University of Newcastle, Callaghan, NSW 2308, Australia

**Keywords:** postures, soccer, sports injury

## Abstract

Posture, a potentially modifiable injury risk factor, is considered important in injury screening/prevention in athletes, yet few studies investigate relationships between posture and injury. This prospective cohort study investigated whether static posture is associated with lower limb injury risk in male football players (*n* = 263). Nine aspects of static standing posture (left and right rearfoot, knee interspace, lateral knee, lumbar lordosis, thoracic kyphosis, scoliosis S and C, forward head) were assessed from photographs during the pre-season using the modified Watson and Mac Donncha scale, which was dichotomised for analysis (deviated or normal). Player characteristics (age, height, mass, body mass index, competition level), match/training exposure, and previous and in-season non-contact lower limb injuries were recorded. Binary logistic regression investigated relationships between posture and injury (previous and in-season). Eighty previous and 24 in-season lower limb injuries were recorded. Previous injury was not associated with any postural variable. In-season injury was associated with previous injury (OR = 3.04, 95% CI 1.20–7.68, *p* = 0.02) and having a normal thoracic curve compared to kyphosis (OR = 0.38, 95% CI 0.15–1.00, *p* = 0.05) but no other postural variables. Static postural deviations observed in male football players in the pre-season are not typically associated with non-contact lower limb injury risk; thus, they are unlikely to add value to pre-season screening programs.

## 1. Introduction

Football (soccer) is one of the most popular sports worldwide [1,2,3,4,5,6,7] with an estimated 270 million players registered across the globe [1] and participation rates growing [8,9]. It requires players to execute player-to-player contact [1,8,10,11], rapid acceleration and deceleration [12], and sharp changes of direction [8]. Participation in football carries a risk of injury for all players independent of skill level or age [1,10], with injuries per 1000 h of total exposure varying in the literature from 2.0 to 44.6 [2,3,10,13] and injuries per 1000 game hours varying from 8.7 to 103.9 [3,10,13,14,15]. This variation may be due to differing definitions of injury and time loss [3,6,13,14,16], various data collection methods [3,14], and different population characteristics in relation to geographical location and skill level [6,13].

Although there is an abundance of literature on injury in professional players [3,15,17], these findings may not be applicable to amateurs [9,11]. Of all football-related injuries, 60–98% are to the lower limbs [1,3,11,14,15,16,17]. Previous studies suggest the most common areas of injury are the ankle, knee, and thigh [2,3,13,15]. Data from one study that investigated injuries in both professional and amateur players suggested amateurs have more injuries to the ankle, whereas professionals have more injuries to the knee [2]. However, another study that included both amateurs and professionals found that the body areas injured were similar. [13] The incidence of injury also varies across previous studies, most likely relating to differences in injury definitions. There are consistently more injuries occurring in matches (e.g., 20/1000 h [11], 33/1000 h [3]) compared to training (e.g., 5/1000 h [11], 9/1000 h [13]). When training and match injuries are combined, the reported incidence rate is lower (9.6 for amateurs, 6.2 professionals) [2]. The overall incidence and severity appears higher in less skilled (amateur) players [13], although one study suggested that amateurs may have more injuries during training, whereas professionals may have more injuries during matches [2]. Nevertheless, an increasing number of amateur players [9], a suggested higher injury incidence rate in lower levels of play [2,13], and the suggestion that amateur players have lower fitness and coordination levels than elite players [9] justifies the need for further investigation of injuries in sub-elite levels of football.

Potential causes or risk factors for injury in football have been proposed, including player-to-player contact [3,9,13,16], previous injury combined with inadequate rehabilitation [9,10,18,19], increased age [1,9,10], leg dominance [7,9], fatigue [1,3], and game compared to training conditions [2,10,13,16]; however, modifiable risk factors in football still require investigation. Posture is one possible contributing risk factor that is potentially modifiable. It has not been widely investigated in football players despite being considered an important aspect of injury screening and prevention in athletes [20]. Of the limited studies investigating posture, increased lumbar lordosis, or extension of the lower back, appears to be associated with increased injury in futsal players [21], hamstring injury risk in rugby, hurling and Gaelic football [22], and a variety of injuries in footballers [23]. Furthermore, in non-sporting populations, increased lordosis has been associated with pars interarticularis fractures [24] and lower back pain [25]. Watson [23] examined static ankle postures in athletes using photographs, observing more abnormal ankle postures in soccer, rugby, and Welsh football players who suffered ankle injuries compared to ankles of uninjured players; however, this association is not consistent with other studies. Nielsen [26], Ramskov [27], and Halabchi [28] reported no significant relationship between injury and foot posture in novice runners [26,27] or elite football and basketball players [28] as measured by the Foot Posture Index, although small sample sizes [27] and low injury counts [26] may have influenced these results. As some static postures (i.e., lumbar and foot postures) have been associated with injury, and there is conflicting evidence regarding the relationships between static postures and injury, further investigation of the relationship between static standing posture and sports injury risk is warranted.

Various methods have been used to assess posture and determine abnormal or deviated postures proposed to be related to injuries. Posture that is considered ‘ideal’ is observed when the centre of gravity of specific body segments is aligned vertically above segments below [29]. A variety of deviations from this alignment can occur, and it is these deviations that are proposed to be related to injury. There are several spinal [30,31,32,33,34,35,36,37] and lower extremity [38] posture measurement techniques that have been reported for objective measurement in laboratory settings. However, these are not practical or feasible for use in a community sporting environment. Watson and Mac Donncha [29] have reported an observational posture scale based on the visual observation of standing photographs that is appropriate for clinical settings and when large populations are assessed. It is the only validated posture scale we identified that encompasses the whole of the body and is feasible for in-field studies. Its methods are consistent with clinical posture assessment that is based on observations. Photographs of posture are scored from 5 (good posture) to 1 (marked deviation). Using a scoring system allows for posture to be quantified so that relationships between posture and injury can be investigated.

Lower limb injuries are common in football [1,14]. Posture is a plausible risk factor that can be assessed in clinical or community sport environments [21,22,23]. If posture deviations were shown to be as associated with increased injury risk, then posture screening could be used as a tool to identify players at risk and implement prevention programs. Therefore, the aim of this study was to determine whether postural deviations are associated with increased risk of lower limb injuries over one competitive season. We hypothesised that postural deviations would be associated with lower limb injury in football. Identifying factors that may predispose players to injury provides opportunities to develop and implement injury screening and prevention programs to improve team success [1,3,6,12], decrease treatment expenses [1,6,15], and decrease the short and long-term impact that injuries may have on individual players’ health [6,15].

## 2. Materials and Methods

In this prospective cohort study, male football players aged 15 years or older were recruited from local club competitions and area-representative teams from a range of competition levels in the Hunter and Central Coast regions of NSW Australia prior to the 2008 and 2009 seasons, with photographic posture data analysed in 2018 using contemporary methods when resources permitted. Competition level was determined as being either high if the player was in a first-grade team (highest non-professional level) or low for any teams below this level. Players were excluded from participating if they displayed signs and symptoms of illness or if they had an injury preventing them from completing pre-season screening. Data on participating players were attained at pre-season screenings and included static posture assessment, standardised anthropometric measures [39] (height, mass, BMI), and player reported age, competition level, and previous lower limb injuries (as reported by the participating player in the pre-season at the data collection session where their posture photographs were taken). Previous injuries were defined as injuries sustained within the previous year resulting in missing 1 or more competition games. Participating players were monitored for non-contact lower limb injuries and training and match exposure during the competition season following their recruitment and screening.

This study was approved by the University of Newcastle Human Research Ethics Committee (H-252-0706). Club and individual player informed consent were provided prior to participation in this study.

### 2.1. Posture Data Collection

Digital photographs of participants were taken using a Pentax, Optic M30 camera, positioned on a tripod at a standardised height, with standardised camera settings for all players. Players stood 3.1 m away from the camera on a small (5.5 cm) box to allow for a clearer view of the feet. Four full body photographs were taken to allow for different views of standing posture: (1) anterior, (2) right lateral, (3) right lateral with arms across chest, and (4) posterior. Players were instructed to stand normally, look straight ahead, and put their feet together if able. Players stood in bare feet and wore shorts only.

### 2.2. Assessment of Posture

**Figure 1:**
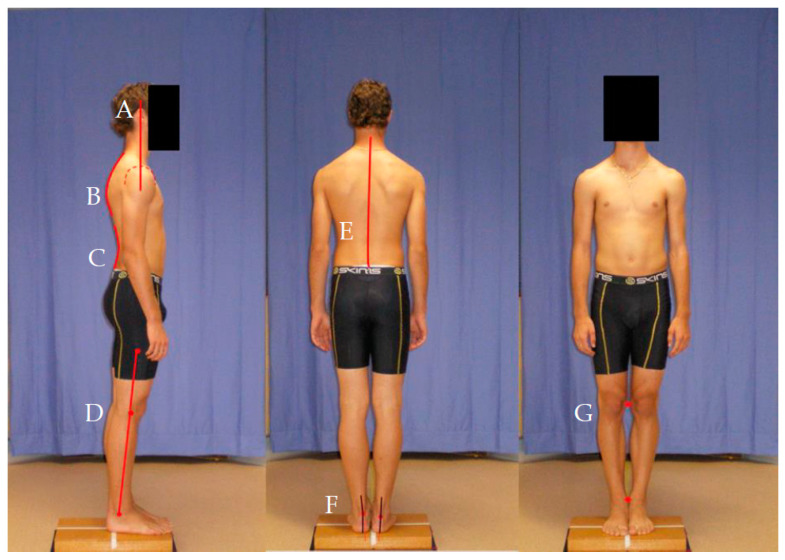
Posture assessment from a typical participant (from left to right): (**A**) Forward head: position of ear in relation to position of midline of shoulder; (**B**) thoracic kyphosis: observed hyperflexion of thoracic spine; (**C**) lumbar lordosis: hyperextension of lumbar spine as per Watson and Mac Donncha [29] scale; (**D**) lateral knee posture: based on vertical alignment of lateral malleolus, midline of knee joint, and greater trochanter. (**E**) Scoliosis C: observed left or right deviation from midline of spine; (**F**) rearfoot posture: angle between a line drawn through Achilles tendon and another through midline of the calcaneus. (**G**) Knee interspace: distances between medial femoral epicondyles and between medial malleoli.

A modified version of the scale created by Watson and Mac Donncha [29] was used to assess the static standing posture of players. From the four photographs, nine aspects of posture were assessed as pictured in Figure 1A–C. Each aspect was scored on a numerical scale from 1 to 5: (1) marked deviation, (2) marked to moderate deviation, (3) moderate deviation, (4) slight deviation, or (5) good posture, i.e., normal or ideal. For all aspects of posture listed below, the diagrams created by Watson and Mac Donncha [29] were used to assist with scoring.

Forward head was assessed by observing the position of the ear in relation to the position of the midline of the shoulder in the horizontal plane. If the ear was anterior to the mid-shoulder, then it was considered forward. Slight to moderate deviation was designated when the ear aligned with the front half or anterior border of the shoulder, and marked deviation was designated when the ear was significantly forward of the anterior border of the shoulder. The presence of scoliosis, or abnormal curvature of the spine in the frontal plane (S and C curve) was assessed by observing the magnitude of left or right deviation from the midline of the spine, and it was categorised as per the diagrams from Watson and MacDonncha [29]. Lumbar lordosis and thoracic kyphosis were assessed based on observing the magnitude of hyperextension of the lumbar spine and hyperflexion of the thoracic spine, respectively. Then, it was determined whether these were normal or excessive as per the diagrams from Watson and MacDonncha [29].

Lateral knee posture was assessed based on the vertical alignment of the lateral malleolus, midline of the knee joint, and the greater trochanter. Deviations from ‘normal’ were described as being hyperflexed or hyperextended. Knee interspace was assessed by observing the distance between the medial femoral epicondyles and determining whether the player deviated into a varus (knee angled outwards) or valgus (knees angled toward midline) alignment. Left and right ankle posture were assessed independently. A line was drawn through the Achilles tendon and another through the midline of the calcaneus. The angle that was created by the intersection of these lines determined whether the player’s rearfoot was deviating from neutral into either inversion (supination) or eversion (pronation). In order to make clinically meaningful inferences, the direction of deviation was assigned for lateral knee posture (hyperflexion or hyperextension), knee interspace (varus or valgus), and ankle posture (varus or valgus).

The reliability of posture scores between two raters was evaluated and determined satisfactory only when posture scores were dichotomised. Thus, lower limb aspects (ankle, knee interspace, lateral knee posture) scored 1–3 were recoded as A, moderate to marked deviation, and those scored 4–5 were recoded as B, slight to no deviation. Spinal aspects (lordosis, kyphosis, scoliosis, forward head) scored 1–4 were recoded as A, any deviation from normal, and those scored 5 were recoded as B, no deviation. Spinal posture was categorised differently to the lower limb as the magnitude of deviation in the spinal posture was difficult to ascertain from photographs, and the piloting of posture assessment indicated that the assessment of different magnitudes of spinal deviation from the photographs was unreliable. This was potentially due to the involvement of multiple joints when assessing spinal posture. The reliability of dichotomised posture scores was determined by repeated posture assessment on a random selection of 20 postures by the same rater for intra-rater reliability (a minimum of one day apart) and by two raters independently for inter-rater reliability (conducted within the same 3-week time frame). All reliability assessments were performed on standard computer monitors using the digital photographs. The two raters were physiotherapy students in the final year of study of their entry-level degree.

### 2.3. Injury and Exposure Monitoring

In the competitive season (approximately 7 months) following the assessment of player posture, team sports trainers and physiotherapists monitored and recorded player injuries. The location of injury was categorised for the lower limb as ankle, calf, knee, quadriceps, hamstring, or groin. Injuries were recorded as either first during the season or recurrent. Only non-contact injuries were included, as contact injuries involve other individuals and would not be expected to be related to static posture. Injuries were included in analyses if they resulted in the player missing a subsequent training session or game. Previous injuries were defined as injuries sustained within the previous year resulting in missing 1 or more competition games.

Team strength and conditioning coaches recorded team-based exposure from the beginning of the pre-season to the end of the competition season via electronic or paper format training diaries. Average exposure hours were calculated for each team as follows: training exposure (number of players in attendance × number of training sessions throughout season), game exposure (number of matches played × duration of matches × number of players), total team exposure (team training exposure + team game exposure), and total exposure per player (total team exposure/number of players). Then, a value for exposure was assigned to each individual player based on the corresponding exposure value per player for their team. Where exposure data for a team were missing (19%, *n* = 51 players), we used exposure data from the same team from a different playing year; where those data were also missing, we used mean exposure data for players of the same age. Exposure was categorised as either high, medium, or low based on the 0–100th percentiles of our data. Low exposure (0–25th percentile) was defined as 0–122 h, medium (26–75th percentile) was defined as >122–166 h and high (≥76th percentile) was defined as >166 h.

### 2.4. Data Analysis

Percent agreement (number of posture responses in agreement as a percent of total number of posture responses for each posture variable) was used to determine inter-rater and intra-rater reliability of the modified Watson and Mac Donncha scale [29]. Sample size calculations were not performed a priori. Descriptive data are reported for player characteristics (age, height, mass, BMI), exposure, performance level, and previous and in-season injury.

Binary logistic regression models were used to determine whether there were associations between previous or in-season non-contact lower limb injury and having one or more postural deviation. Relationships between specific injury categories (ankle, knee, hamstring, quadriceps groin, and calf) and potentially related posture deviations (e.g., rearfoot, knee interspace, and lateral knee posture for ankle injuries) were explored with univariate binary logistic regression. To increase power due to the small number of non-contact injuries recorded in each category, non-contact lower limb injuries as a whole were analysed for associations with postural variables. Separate models were used for previous and in-season injury, and all postural deviations were explored. The following potential confounders were investigated: player age, height, mass, BMI, performance level and exposure as well as previous injury for the in-season injury model. Due to the large number of variables, univariate regressions were first performed for both previous and in-season injury, with only those variables achieving a *p* ≤ 0.25 in univariate analysis being included in the multivariate models [40]. As a separate analysis, the relationship of BMI (categorised as underweight < 18.5, healthy 18.5 to < 25, and overweight/obese ≤ 30) to each posture variable was explored using Chi-square to determine whether it should be considered for the multivariate models. Final models were determined using the backwards Wald method with either previous or in-season injury as the dependent variable and postural variables or confounders as the independent variables. Both of the final models were also examined with exposure and competition level included as independent variables because of the potential these factors have to an influence on the injury risk. All analyses were performed in IBM SPSS Version 24.0 (IBM Inc., Armonk, NY, USA).

## 3. Results

Descriptive statistics of player characteristics and injury are displayed in Table 1. A total of 80 previous and 24 in-season lower limb injuries were recorded in the sample of 263 players.

The majority of players were categorised into the medium exposure group (62.0%, *n* = 163) and were considered to be participating at a low competition level (68.1%, *n* = 179). The most common postural deviations were forward head (60.8%, *n* = 160), thoracic kyphosis (52.9%, *n* = 139), and lumbar lordosis (39.9%, *n* = 105). Only one player had scoliosis with an S-curve; therefore, scoliosis S was excluded from further analyses.

The modified Watson and Mac Donncha scale [29] demonstrated high inter-rater and intra-rater reliability, as displayed in Table 2. Across all the posture areas, intra-rater reliability ranged from 80 to 100%, and inter-rater reliability ranged from 75 to 100%. Postural deviations for the 24 players with in-season injuries are reported in Table 3, with the type of in-season injury sustained and the type of previous injury if applicable.

The univariate models for previous and in-season non-contact injuries as a whole demonstrated that age, mass, BMI, exposure, and competition level were not associated with having previous or in-season injuries (Table 4 and Table 5) and therefore, these covariates did not meet our criteria for inclusion in the final models. Height demonstrated an association with previous injury, but not in-season injury, and it was therefore included in the final model for previous lower limb injury. Previous lower limb injury was associated with in-season injury (*p* = 0.03) and met the criteria for inclusion in the final model for in-season injuries. The categorised BMI variable was associated with one posture variable, thoracic kyphosis. A greater proportion of players in the underweight category (10 kyphotic players vs. 1 normal) and a lower proportion in the overweight/obese category (13 players vs. 22) had kyphotic posture. However, the large majority of players had normal thoracic posture. Categorised BMI was not associated with injury in univariate models so did not meet the criteria for inclusion in the final multivariate models.

The final regression model for previous lower limb injury demonstrated that there was no significant association with any postural variable. The final regression model for in-season lower limb injury demonstrated that there was no association with the majority of postural variables. Thoracic posture was the only postural variable that showed a statistically significant association with in-season lower limb injury (Table 6). Having a normal thoracic posture, as compared to having kyphosis, was associated with lower limb injury. The inclusion of exposure and competition level in either final model did not alter the effect of postural deviation on injury risk, and therefore, models are reported without these potential confounders. The final model for in-season injury included previous injury, as this was significant when combined with the thoracic posture variable.

## 4. Discussion

The main finding of this study was that players with a normal thoracic kyphosis were more likely to have a lower limb injury during the playing season, which is consistent with findings from Lotfian et al. [41]. This study also found that spinal postural deviations, i.e., forward head, thoracic kyphosis, and lumbar lordosis, were common in amateur football players. However, there were fewer players with postural deviations of the lower limbs, which might be expected to have an association with lower limb injury. A lack of thoracic kyphosis, or a more normal posture, was associated with increased injury risk. While the reason for this finding cannot be confirmed, it is possible that those players with more normal posture were perhaps more active in general outside of football, increasing their additional exposure to injury [42] that was not captured in team exposure, or played more aggressively [43], placing them at greater risk. However, in the current study, exposure and competition level were not shown to affect injury risk nor the relationship between posture and injury risk. It is possible that additional factors not collected in the current study such as training or game conditions may influence injury risk and mask the effect of posture. Thus, the multi-faceted nature of sporting injuries should be considered in future studies that investigate posture.

The association between in-season injury and having a normal thoracic posture, compared to kyphosis, in the current study is consistent with a study conducted by Lotfian et al. [41]. Lotfian et al. found increased thoracic kyphosis to be potentially protective against injury, with a greater thoracic kyphosis Cobb angle being associated with a reduction in the likelihood of major quadriceps injury [41]. The thoracic spine Cobb angle is the acute angle formed by the intersection of two lines: one drawn through the two superior vertebral body corners of T1 and the other through the two inferior vertebral body corners of T12, which was traditionally measured with radiographs. [44] Lotfian et al. used a Spinal Mouse device to quantitatively measure the thoracic spine Cobb angle, whereas the current study used observations from photographs. Nonetheless, their findings are consistent with the finding from the current study that having thoracic kyphosis does not increase vulnerability to injury. Interestingly, the current study found having a lower (underweight) BMI was associated with kyphotic posture. However, BMI was not associated with injury and did not affect the relationship between posture and injury. Lotfian et al. also found a lack of association between lower limb injury and cervical rotation range of motion [41] measured in degrees. While this measurement differs from the observation of forward head posture in the current study, both studies found a lack of relationship between head/neck-related variables and lower limb injury. Previous studies using similar observational methods to the current study have reported an association between hyper-lordosis of the lumbar spine and injury in sporting populations [21,22,23]; however, this was not supported in the current study, which found no association between lumbar spine posture and injury.

In the current study, rearfoot posture was not related to lower limb injury, which is a finding that is consistent with some previous literature that utilised the Foot Posture Index, a validated measure of foot posture, to classify foot position [26,27,28]. This is contradictory to Watson’s study, which found more abnormal ankle postures in sports people who suffered ankle injuries when using an observational method similar to the current study [23]. Together, these findings suggest that static posture is unlikely to have a strong relationship with injury risk. However, considering the conflicting evidence, further evaluations of posture in relation to sporting injuries are warranted. Future studies should consider the impact of different methods of assessing posture and defining injury, as these differed substantially between studies.

A low number of in-season injuries (*n* = 24) were sustained during this study. This may be a result of the healthy, young population being studied, as increased age is associated with increased injury risk [1,8,10,14,19]. It is also possible that low injury rates may be due to the lower exposure hours [2,13] and intensity of play of amateur players in comparison to professionals, although this is contrary to the literature suggesting that injury rates in amateur footballers are higher [2,11,13]. The weak association between previous and in-season injury approaching significance (*p* = 0.055), which is consistent with what has been established previously in the literature [9,10,18,19], may be due to the low proportion of in-season injuries in the current study. Age, height, mass, BMI, exposure, and competition level were not associated with injury. These variables had low variation within the sample, as players were of similar ages and playing levels, thus making it difficult to detect an effect.

Several limitations should be considered when interpreting the results of this study. Collecting injury and exposure data in an amateur environment is challenging. Players are not obliged to report injuries, they may play additional sports outside the target study increasing their exposure, and teams may not have regular staff to record exposure. Additionally, defining injuries via time loss from football-related activity may mean that some less severe injuries are not recorded at the amateur level, which may be captured in professional athletes that are training every day. Another limitation is the evaluation of static posture from photographs only rather than in-person assessment by a health professional, and using observation without specific anatomical measurements. Although commonly assessed in athletes, static posture may not be functionally related to football and only provides one piece of a larger puzzle that explains athletes’ injury risk. A small number of posture deviations in the lower limbs were observed, which may have limited our ability to observe significant associations between posture and lower limb injury.

The strengths of this study should also be acknowledged. The modified Watson and Mac Donncha scale [29] was rigorously tested for reliability, and modifications were used to maximise its reliability. The scale is an assessment tool that can realistically and easily be applied to an amateur sporting environment and does not require extensive training of raters or access to expensive equipment. In the current study, this method was able to detect a large number of postural deviations, particularly of spinal posture. Supporting this, computer-based photographic posture assessment has been reported as useful and easy to use by clinicians [45]. Photographic assessment tools, such as the scale used in the current study, have the potential to facilitate posture analysis and to improve patient education regarding their musculoskeletal impairments; however, the additional time needed to use such tools in the clinic or sport setting should be considered [45]. Other strengths of the study are the relatively large sample size for a study that includes clinical assessment, the number of posture variables included, and the inclusion of playing exposure and past injury as potential confounding variables.

## 5. Conclusions

This study showed that the modified Watson and Mac Donncha scale can be reliably used in amateur sporting environments with young players. However, other than thoracic posture, variables of static standing posture as assessed by this scale in the current study were not associated with previous or in-season non-contact lower limb injuries in amateur football players. This study was limited by low numbers of lower limb injuries, and the majority of posture deviations occurred in the spine. Therefore, further research on the relationship between static posture and injury may be warranted. Based on the findings of the current study, assessment of static posture in the pre-season is not recommended for determining if a player is at risk of non-contact lower limb injury. Further research into posture should potentially include additional factors that may contribute to injury risk in young football players.

## Figures and Tables

**Table 1:** Characteristics of male amateur football players included in the study (*n* = 263), those with a previous lower limb injury (*n* = 80), and those with a non-contact in-season lower limb injury (*n* = 24).

Characteristic	All Players (*n* = 263)	Previous Injury	In-Season Non-Contact Injury
Age (y), mean (SD)	18.3 (3.3)	17.8 (3.1)	17.8 (2.5)
Height (cm), mean (SD)	176.9 (5.9)	175.9 (5.7)	175.8 (6.8)
Mass (kg), mean (SD)	70.1 (10.2)	68.9 (9.7)	69.3 (10.2)
BMI (kg/m^2^), mean (SD)	22.3 (2.6)	22.2 (2.4)	22.4 (2.8)
Exposure (h), number (%) Low (0–122)	37 (14.1)	12 (15.0)	2 (8.3)
Medium (123–166)	163 (62.0)	52 (65.0)	16 (66.7)
High (>166)	63 (24.0)	16 (20.0)	6 (25.0)
Competition Level (category), number (%) Low	179 (68.1)	56 (70.0)	16 (66.7)
High	84 (31.9)	24 (30.0)	8 (33.3)
Previous Injury (category), number (%) Any	80 (30.4) *	80 (100) ^†^	11 (13.8) ^†^
Ankle	31 (11.8)	31 (38.8)	4 (5.0)
Knee	19 (7.2)	19 (23.8)	3 (3.8)
Hamstring	13 (4.9)	13 (16.3)	1 (1.3)
Quadriceps	9 (3.4)	9 (11.3)	2 (2.5)
Calf	11 (4.2)	11 (13.8)	1 (1.3)
Groin	10 (3.8)	10 (12.5)	2 (2.5)
In-Season Injury (category), number (%)Any	24 (9.1) *	11 (47.8) ^†^	24 (100) ^†^
Any ankle	10 (3.8)	1 (4.3)	10 (41.7)
Any knee	7 (2.7)	3 (13.0)	7 (29.2)
Any hamstring	3 (1.1)	4 (17.4)	3 (12.5)
Any quadriceps	4 (1.5)	0 (0)	4 (16.7)
Any calf	3 (1.1)	3 (13.0)	3 (12.5)
Any groin	3 (1.1)	1 (4.3)	3 (12.5)

* Percent within cohort, individual players may have had more than one body area injured, ^†^ Percent within category.

**Table 2:** Percent agreement between repeated measurements of posture (*n* = 20) for a single rater (intra-rater) and two raters (inter-rater).

Postural Aspect	Intra-Rater N/20 Agreed on *(%)*	Inter-Rater *N/20 Agreed on (%)*
Left rearfoot	17 (85)	17 (85)
Right rearfoot	16 (80)	15 (75)
Knee interspace	19 (95)	17 (85)
Lateral knee posture	18 (90)	19 (95)
Lumbar lordosis	19 (95)	17 (85)
Thoracic kyphosis	16 (80)	16 (80)
Scoliosis C	20 (100)	19 (95)
Scoliosis S	20 (100)	20 (100)
Forward head	20 (100)	18 (90)

**Table 3:** Postural deviations * in injured players (*n* = 24), with their type of in-season injury, and if applicable, their previous injury.

Lower Limb in-Season Injury	Knee Interspace	Lateral Knee	Lordosis	Kyphosis	Scoliosis C	Scoliosis S	Forward Head	Previous Injury
Ankle sprain							X	Knee strain
Ankle sprain	X (varus)		X					
Ankle sprain							X	
Ankle sprain							X	Hamstring strain
Ankle sprain								
Ankle sprain		X (extension)		X			X	Ankle sprain
Ankle sprain				X			X	Ankle sprain, calf strain
Ankle sprain, hamstring strain	X (varus)		X				X	
Ankle sprain, hamstring strain		X (flexion)		X				
Ankle sprain, knee sprain	X (varus)			X			X	
Knee sprain				X			X	Calf strain
Knee sprain			X		X		X	Calf strain
Knee sprain							X	
Knee sprain							X	
Knee sprain			X					Hamstring strain
Knee sprain, calf strain			X				X	
Quad strain							X	Knee strain
Quad strain	X (varus)		X				X	
Quad strain								
Quad strain, groin strain	X (varus)		X				X	Hamstring strain, calf strain
Groin strain				X			X	Groin strain
Groin strain, calf strain				X			X	
Hamstring strain				X			X	Hamstring strain
Calf strain							X	Knee sprain

**Table 4:** Univariate regression analysis for factors potentially associated with previous injuries in football players (*n* = 263), with descriptive statistics for each variable examined.

Variable	N in Categories N (%)	N Players Injured in Each Group N (%)	OR (95% CI)	*p*-Value
Age (y) *	Not injured: 18.2 (3.1) Injured: 17.8 (3.1)	-	0.96 (0.86–1.06)	0.37
Height (cm) *	Not injured: 177.0 (5.8) Injured: 175.9 (5.7)	-	0.96 (0.92–1.01)	0.13
Mass (kg) *	Not injured: 70.0 (10.1) Injured: 68.9 (9.7)	-	0.99 (0.96–1.02)	0.46
BMI (kg/m^2^) *	Not injured: 22.3 (2.7) Injured: 22.2 (2.4)	-	1.0 (0.89–1.11)	0.93
Exposure				
Low (ref) 0–122 h	37 (14.1)	12 (32.4)	1	0.72
Medium 123–166 h	163 (62.0)	52 (31.9)	1.46 (0.56–3.77)	0.44
High > 166 h	63 (24.0)	16 (25.4)	1.08 (0.55–2.15)	0.82
Competition Level			1.02 (0.57–1.85)	0.76
Low	179 (68.1)	56 (31.3)		
High	84 (31.9)	24 (28.6)		
Left rearfoot posture			0.79 (0.20–3.14)	0.74
Deviated	11 (4.2)	3 (27.3)		
Not deviated	252 (95.8)	77 (30.6)		
Right rearfoot posture			1.96 (0.71–5.43)	0.20
Deviated	17 (6.5)	8 (47.1)		
Not deviated	246 (93.5)	72 (29.3)		
Knee interspace			0.85 (0.45–1.61)	0.61
Deviated	61 (23.2)	18 (29.5)		
Not deviated	202 (76.8)	62 (30.7)		
Lateral knee posture			0.70 (0.28–1.75)	0.44
Deviated	26 (9.9)	7 (26.9)		
Not deviated	237 (90.1)	73 (30.8)		
Lordosis			0.82 (0.47–1.42)	0.48
Deviated	105 (39.9)	31 (29.5)		
Not deviated	158 (60.1)	49 (31.0)		
Kyphosis			1.41 (0.82–2.44)	0.22
Deviated	139 (52.9)	46 (33.1)		
Not deviated	124 (47.1)	34 (27.4)		
Scoliosis C			0.76 (0.26–2.24)	0.62
Deviated	17 (6.5)	5 (29.4)		
Not deviated	246 (93.5)	75 (30.5)		
Forward head			1.22 (0.70–2.14)	0.49
Deviated	160 (60.8)	51 (31.9)		
Not deviated	103 (39.2)	29 (28.2)		

* Mean (SD) reported for these variables.

**Table 5:** Univariate regression analysis for factors potentially associated with in-season lower limb non-contact injuries in football players (*n* = 263), with descriptive statistics for each variable examined.

Variable	N in Categories (%)	N Players Injured in Each Group (%)	OR (95% CI)	*p*-Value
Age (y) *	Not injured: 18.3 (3.3) Injured: 17.8 (2.5)	-	0.5 (0.81–1.12)	0.56
Height (cm) *	Not injured: 177.0 (5.8) Injured: 175.8 (6.8)	-	0.97 (0.90–1.04)	0.37
Mass (kg) *	Not injured: 70.1 (10.2) Injured: 69.3 (10.2)	-	0.99 (0.95–1.04)	0.69
BMI (kg/m^2^) *	Not injured: 22.3 (2.6) Injured: 22.4 (2.8)	-	1.01 (0.85–1.18)	0.96
Exposure			1	0.70
Low (ref) 0–122 h	37 (14.1)	2 (5.4)		
Medium 123–166 h	163 (62.0)	16 (9.8)	0.54 (0.10–2.84)	0.47
High > 166 h	63 (24.0)	6 (9.5)	1.03 (0.39–2.77)	0.95
Previous lower limb injury			2.75 (1.10–6.83)	0.03
Injured	80 (30.4)	12 (15.0)		
Not injured	149 (56.7)	9 (6.0)		
Competition Level			1.07 (0.44–2.62)	0.88
Low	179 (68.1)	16 (8.9)		
High	84 (31.9)	8 (9.5)		
Left rearfoot posture			_	1.0
Deviated	11 (4.2)	0 (0)		
Not deviated	252 (95.8)	24 (9.5)		
Right rearfoot posture			_	1.0
Deviated	17 (6.5)	0 (0)		
Not deviated	246 (93.5)	24 (9.5)		
Knee interspace			0.86 (0.31–2.41)	0.77
Deviated	61 (23.2)	5 (8.2)		
Not deviated	202 (76.8)	19 (9.4)		
Lateral knee posture			0.81 (0.18–3.68)	0.79
Deviated	26 (9.9)	2 (7.7)		
Not deviated	237 (90.1)	22 (9.3)		
Lordosis			0.59 (0.24–1.48)	0.26
Deviated	105 (39.9)	7 (6.7)		
Not deviated	158 (60.1)	17 (10.8)		
Kyphosis			0.41 (0.17–1.00)	0.05
Deviated	139 (52.9)	8 (5.8)		
Not deviated	124 (47.1)	16 (12.9)		
Scoliosis C			0.61 (0.08–4.78)	0.64
Deviated	17 (6.5)	1 (5.9)		
Not deviated	246 (93.5)	23 (9.3)		
Forward head			2.64 (0.95–7.31)	0.06
Deviated	160 (60.8)	19 (11.9)		
Not deviated	103 (39.2)	5 (4.9)		

* Mean (SD) reported for these variables.

**Table 6:** Final multivariate model for in-season non-contact lower limb injuries.

Variable	N in Categories (%)	N Players Injured in Each Group (%)	OR (95% CI)	*p*-Value
Previous injury			3.04 (1.20–7.68)	0.02
Injured	80 (30.4)	11 (13.8)		
Not injured	149 (56.7)	9 (6.0)		
Kyphosis			0.38 (0.15–1.00)	0.05
Deviated	139 (52.9)	7 (5.0)		
Not deviated	124 (47.1)	16 (12.9)		

## Data Availability

The data presented in this study are available on request from the corresponding author. The data are not publicly available due to restrictions, i.e., they contain information that may compromise the privacy of research participants.
